# Respirator fit of a medium mask on a group of South Africans: a cross-sectional study

**DOI:** 10.1186/1476-069X-10-17

**Published:** 2011-03-15

**Authors:** Adri Spies, Kerry S Wilson, Robert Ferrie

**Affiliations:** 1 University of the Witwatersrand, School of Public Health, Private Bag 3, Wits 2050, South Africa; 2 National Institute for Occupational Health, PO Box 4778, Johannesburg, 2000, South Africa; 3 OHS Consultant, PO Box 423, Douglasdale, 2165, South Africa

## Abstract

**Background:**

In South Africa, respiratory protective equipment is often the primary control method used to protect workers. This preliminary study investigated how well a common disposable P2 respirator fitted persons with a range of facial dimensions.

**Methods:**

Quantitative respirator fit tests were performed on 29 volunteers from different racial, gender and face size groups. Two facial dimensions width (bizygomatic) and length (menton-sellion) were measured for all participants.

**Results:**

In this study 13.8% of the participants demonstrated a successful fit with the medium sized mask. These included participants from three different racial and both gender groups. The large percentage of failed fit tests (86%) indicates that reliance on off-the-shelf respirators could be problematic in South Africa.

**Conclusions:**

The limitations of this preliminary study notwithstanding, respirator fit appear to be associated with individual facial characteristics and are not specific to racial/ethnic or gender characteristics.

## Background

Personal protective equipment (PPE) is widely used to protect workers from exposure to airborne hazardous substances although it should be viewed as a last resort after other control methods have been implemented. Unfortunately, it is often the only "control" method used in many South African workplaces and the amount of protection provided is likely to be influenced by the fit to the wearer's face. Therefore it is essential that the respirator is properly fitted.

A quantitative fit test is the most accurate way to assess whether a specific type, model and size of respirator adequately fits a particular individual. This involves measuring the concentration of a contaminant inside and outside a respirator and expressing the ratio as a quantitative fit factor. A half-mask respirator, which fits correctly, must obtain a minimum fit factor of 100, based on Occupational Safety and Health Administration (OSHA) regulations, USA [1]. Equally important, fit testing assists an individual to know how to don and wear the respirator properly [2,3].

People come in many shapes and sizes and therefore the ability of a given respirator to form an effective barrier between the wearer and the contaminated environment may be affected by facial characteristics [4-6]. When the respirator-user fit is not checked, an unsatisfactory seal may unknowingly exist. This will allow leakage of airborne contaminants into the wearer's breathing zone, even though the worker is wearing the correct respirator for the application. This can put the workers at risk of adverse health effects.

The design of respirators is based on anthropometric (human facial size and shape) data obtained from groups of people in respirator fit test panels (RFTPs) [4]. The current respirator fit test panels commonly used to design respirators were originally developed by the National Institute for Occupational Safety and Health (NIOSH) and are based on a facial anthropometric survey of USA Air Force personnel [7].

Findings from a USA study recommended face length and width measurements for defining the RFTPs for half-face respirators [5]. Facial dimensions are likely to be different for various ethnic groups so the applicability of the RFTPs needs to be confirmed as past studies on respirator fit have mostly been carried out on Caucasian and/or male subjects with little attention paid to other races or females [5].

A subsequent study investigated whether American RFTPs are applicable to China's workforce. The results showed that 12-35% fell outside the ranges derived from American RFTPs as Chinese subjects generally had shorter and wider facial characteristics than American groups [4].

Results of a similar Korean study showed that Korean males and females have different facial dimensions compared with those of white American males and females. It was found that a better respirator fit could be achieved by more males than females, regardless of respirator brands tested [6].

Training in respirator donning and fit testing is inseparable. In New Orleans, an investigation evaluated the correct donning of a N95 filtering face-piece respirator. These respirators were used during post-hurricane mould remediation. Only 24% of the 538 participants had donned the respirators properly, without prior training. Errors made by the participants during the study included: nose clip not tightened (71%); straps incorrectly placed (52%); and placing the respirator upside down (22%) [2].

The use of quantitative fit testing to ensure that a respirator fits properly is very rare in South Africa. This preliminary study investigated how well a commonly-used medium mask fitted a group of 29 South Africans of different gender and race and with a range of facial dimensions to determine a respirator fit factor.

## Methods

In this preliminary cross-sectional study quantitative respirator fit tests were performed on 29 volunteers employed at a single workplace (a multi-discipline research organization) during the study period. Subjects were randomly selected and included a range of face lengths and widths that ensured participants covered all four face groups derived from the NIOSH fit test panel [7]. Participants were from all race groups and both genders. Inclusion criteria were that each participant signed informed consent to participate. Permission to conduct this study was obtained from the University of the Witwatersrand Ethics Committee (Number: R14/49 Spies).

**Figure 1:**
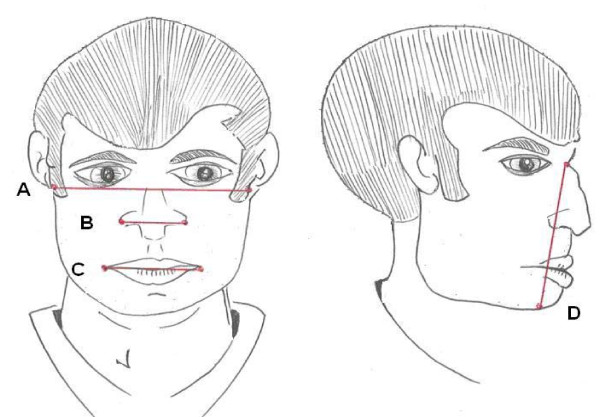
**Diagram of key facial measurements**.

The measurements were made with an anthropometric sliding caliper. Face length was measured from the sellion to the menton (Figure 1D). The placement of these two points was confirmed with finger palpation prior to measurement. Face width was measured between the left and right zygomatic arches and again these were located with finger palpitations (Figure 1A). Lip length was measured from one corner of the mouth to the other while the participant was relaxed (Figure 1C). The nasal width was measured at the widest point of the nose (Figure 1B) in a small selection of participants.

### Face Groups

**Figure 2:**
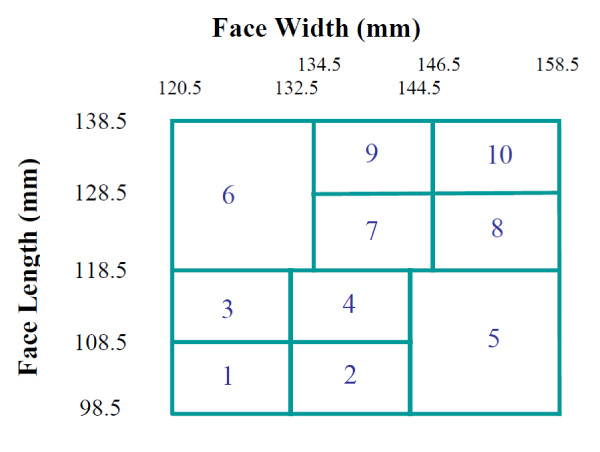
**Face groups of study participants**.

Four face groups were initially defined based on the face measurements taken. These were grouped into small, medium and large based on the NIOSH bivariate panel as follows:

Cells 1-3 as small, cells 4-7 medium and cells 8-10 large (Figure 2)[7].

### Respirator

Each person wore a new medium-sized disposable P2 particulate respirator of the same brand generally available in South Africa.

### Fit testing

A TSI Portacount Plus with N95 Companion was used to conduct the quantitative fit testing and provide a numerical measure of the 'fit factor', an objective measure of face fit. A successful fit requires a fit factor of 100 for half-face respirators and disposable masks. Fit factor means a quantitative estimate of the fit of a particular respirator to a specific individual, and typically estimates the ratio of the concentration of a substance in ambient air to its concentration inside the respirator when worn.

This quantitative fit test is a direct objective measurement of the respirator face seal performance and the procedure (Table 1) as described by the Occupational Safety and Health Administration (OHSA), USA was followed [1].

**Table 1:** The OSHA Ambient Aerosol (PortaCount) Quantitative (QNFT) Protocol

Exercise/Activity	Time (min)
1 Normal breathing	1:00
2 Deep breathing	1:00
3 Turning head side to side	1:00
4 Moving head up and down	1:00
5 Talking	1:00
6 Grimace	0:15
7 Bending over	1:00
8 Normal breathing	1:00

	**Total 7:15**

Each of the participants was fit tested twice. A pass was considered a fit factor of greater than or equal to100 for one of the fit tests. Before each fit test conducted the participants were training in the correct donning of a respirator.

The respirator shall not be adjusted once the fit test exercises begin. Any adjustment voids the test, and the fit test must be repeated. Participants donned the respirator and were examined for correct placement of the respirator. They completed the test in one sitting.

### Statistical analysis

Data were collated in the Excel software program and exported to STATA 9 for analysis. The face width and length were described using standard descriptive statistics and the results of the repeat fit tests were averaged to provide the final fit factor. The fit factor was a continuous variable which was converted into a binomial variable using the cut off for fit of 100. This binomial was used to divide the participants into two groups; those that pass and those that fail. Correlation between both face width and face length was performed. A Student t-test was used to compare fit test results between gender and race groups and between studies.

## Results

This study randomly recruited 29 - employees. This process was repeated until the study group included all race groups and both genders. Standard face groups described above were used to group volunteers. The study population included four small faces, 21 medium faces, three large faces and one outside.

Only Coloured and Asian races were underrepresented in this study due to the small proportion of these groups in the general South African population (Table 2). According to the mid-2007 estimates from Statistics South Africa, Africans are in the majority at just over 38-million, making up 79.6% of the total population. The white population is estimated at 4.3-million (9.1%), the coloured population at 4.2-million (8.9%) and the Indian/Asian population at just below 1.2-million (2.5%) [8].

**Table 2:** Distribution of participants by gender and race

	*African*	*European*	*Coloured*	*Asian*
Male n (%)	8 (28)	4 (14)	2 (7)	0

Female n (%)	5 (17)	8 (28)	0	2 (7)

Table 3 shows the mean facial dimensions of participants in this study compared to previously published dimensions from Korea and the USA [6,9]. The male facial dimensions from this study were found to be significantly different from those measured in Americans (p < 0.001) but not significantly different from the Koreans [6]. Due to the small number of lip and nose widths measured in our study, these were not compared between studies (see Table 3*). This group included all participants who had a successful fit test and others with similar face sizes.

**Table 3:** Comparison of mean face dimensions between South Africa, Korea and USA

*Face Dimensions*	*South African* *this study*		*Korean - Kim et al., 2003*		*American - Oestenstad and Perkins, 1992*	
	
	Male n = 14	Female n = 15	Male n = 70	Female n = 40	Male n = 38	Female n = 30
Face width (mm) Mean (SD)	150.3 (6.6)	141.9 (7.9)	147.6 (5.0)	136.6 (4.9)	139.0 (8.0)	129.0 (6.0)
Face length (mm) Mean (SD)	117.9 (8.3)	111.7 (6.6)	120.6 (5.9)	109.6 (4.2)	126.0 (7.0)	118.0 (5.0)
Lip width (mm) Mean (SD)	50.9*	53.5*	49.3 (3.8)	44.1 (3.2)	51.0 (4.0)	48.0 (3.0)
Nose width (mm) Mean (SD)	41*	40*	36.7 (2.7)	33.2 (1.9)	36.0 (3.0)	33.0 (4.0)

### NIOSH fit test panel (RFTP)

**Figure 3:**
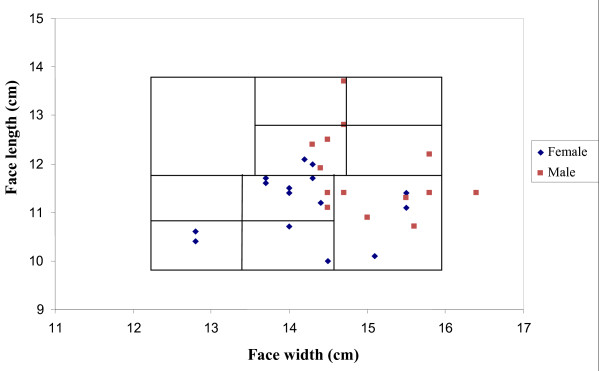
**Scatter plot of South African participants against the improved NIOSH respirator fit test panel (RFTP) **[7].

Overlaid on the scatter plot of participants' facial characteristics is the current NIOSH RFTP (Figure 3). This panel has been shown to include 95% of American study subjects found mainly in the central blocks [7]. This panel also included 95% of South African participants. However this panel may not be an ideal fit as in our study group the majority of participants are found in the bottom right blocks rather than spread across all the blocks. In this study the female face dimensions were significantly different from the males. Male face lengths were significantly longer (p = 0.03) and face widths significantly wider (p = 0.005). Figure 3 shows a scatter plot of the combination of face width and length which remains significantly different for men and women (p = 0.0005). There was no correlation between face width and face length (r = 0.0919 p = 0.6353) for the study group as illustrated in Figure 3.

Fit factor did not differ significantly by gender with a mean difference of 1.2 (p = 0.9830) despite significant differences in the facial dimensions (Table 4). There was variation in the mean fit test results by race but with large standard deviations it is likely that the small numbers in some of the groups caused these differences.

**Table 4:** Mean fit factor across gender and race group

	African	White	Coloured	Asian
Male mean (SD) median	22.1 (18) 16. 5	41.8 (60) 13.5	89.5 (102) 89.5	N/A

Female Mean (SD) median	70.6 (89) 12	5.6 (16) 21	0	0 (4.2) 10

**Figure 4:**
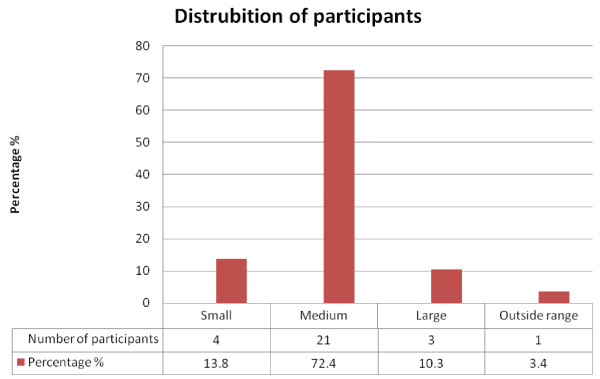
**The study participants' face group sizes**.

The majority of study participants when placed into face groups based on their face width and length measurements fell into the short-wide (42%) and large (46%) groups (Figure 4).

**Figure 5:**
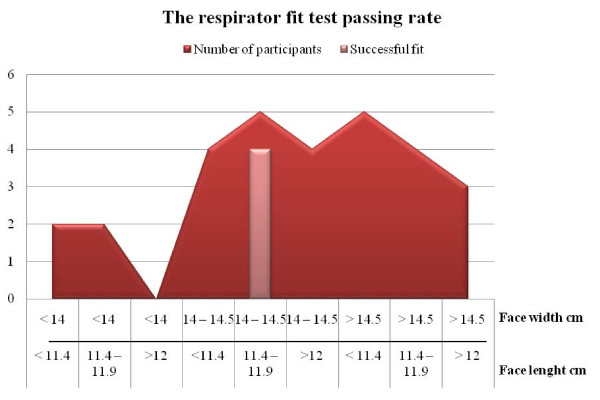
**The study participants' successful respirator fit test and facial dimensions**.

Only 13.8% (n = 4) of the participants demonstrated a successful fit (fit factor >100) (Figure 5). These included participants from three different racial and both gender groups. These participants who had a successful fit test had a limited face width range (14.0 - 14.5 cm) and a limited face length range (11.4-11.9 cm).

One participant who failed the fit test was investigated further by measuring dimension that could play a role in fit testing such nose width. A small selection of participants was included for comparison.

The selected participants in Table 5 had a limited face width range (14.0 - 14.5 cm) and a limited face length range (11.4-11.9 cm). Of the five participants who fitted into these face dimension ranges, four passed the fit test while the fifth did not. The fifth participant had a significantly smaller nose width of 2.9 cm (p = 0.01).

**Table 5:** Selected participants' nose and mouth widths

	Nose width(cm)	Lip Width (cm)
Passed (n = 4)	4.1	5.1

Failed (n = 3)	4.0	5.5

## Discussion

This preliminary study defined the performance of a commonly available medium half mask respirator against facial dimensions from a South African population. American military personnel anthropometric facial values are commonly used when respirators are designed [10]. However studies on respirators designed using these NIOSH RFTPs have mostly focused on white (Caucasian) males [7,11]. Until recently, only a small number of studies included other ethnic groups, mainly Asian [4,12] and these studies illustrated that other ethnic groups were afforded a lower level of protection than white males when using the same respirators [13].

Studies of Chinese and Korean workers showed that facial anthropometric measurements were significantly different from those of American groups. This suggests that the NIOSH American RFTPs may not fairly represent the facial anthropometric characteristics of these Asian groups as the latter had shorter and wider faces [6,14]. These facial characteristic differences are similar to those found in the present study on South Africans, where most participants (irrespective of race) had medium (Figure 4) and did not appear to fit well into the NIOSH RFTPs. This lack of fit may be due to the small number of participants.

This study demonstrated a significant lack of fit of a medium half face respirator across all race groups and both genders. 13.8% of the participants' masks demonstrated a successful face fit. The medium-sized respirator fitted a much smaller proportion of the sampled population than expected.

Those participants with a successful fit test fall within a narrow range of both face width and length measurements (Figure 5). Participants who had only one measurement in these narrow ranges did not produce a successful fit, indicating the complexity of predicting good fit. Thus, placement within the range identified for both face width and length may be a predictor of good fit. There was one participant in the study who fell within the narrow ranges of the length and width but failed the fit test. Further investigation indicated that the nasal width of this participant was significantly smaller than the rest of the group who had a successful fit. This suggests that face width and length may predict fit test results as long as all other facial dimensions are within normal ranges.

This study's group dimensions were based on the newly developed NIOSH RFTPs [7]. In Figure 4 a small number of participants had small or large face sizes, while a large number of participants (72%) had medium face sizes. The distribution of the medium faces in the bivariate panel may play a role in the poor fit demonstrated by a medium respirator.

Gender was found to play a significant role in the measured facial dimensions with men and women significantly different in their measurements. This did not translate to respirator fit as there was no significant difference in fit test results between men and women. This finding corresponds well with the results of a recent Oestenstad study [15] where the effect of gender on facial characteristics was found not to play a significant role in respirator fit. Oestenstad's study did identify that facial dimensions other than face length and width may also be significantly associated with respirator fit suggesting that these other dimensions need to be included in respirator design or used to choose the correct respirator size.

No significant correlation was found in our study between face width and length. This may reflect the mixed heritage of participants and the relationship between their facial dimensions. This finding does not correspond with the findings of other studies like Kim and Oestenstad [6,15] which suggests that both these studies were conducted on more homogeneous populations and may partly account for the finding of poor respirator fit for the majority of our study population.

The current study correlation analysis was not performed between the facial dimensions and fit factor. Also linear regression was not applicable to the current study as the relationship between fit factor and facial dimensions was not linear.

In our study there were a number of limitations. Firstly, the representation of the small study group to the general South African workforce cannot be determined. The study did include faces from all three NIOSH face groups so it can give an expectation of results from a larger representative work force. Secondly, the study only measured a limited number of facial characteristics, which have been suggested elsewhere as related to respirator fit [6,9]. The representation of race groups was not equal and may have introduced some selection bias. The use of one specific type of respirator does limit the fit in small and large face groups but it is representative of South African workplaces where medium respirators are often the only type available essentially for cost-effectiveness reasons.

The poor fit measured for most of our study subjects suggests that the common use of a medium respirator in South African workplaces may place workers at risk of harmful exposures. This can have a significant implication for the health of the worker. The results of this study need to be tested in a larger more representative sample of South African workers. This larger sample could also provide a description of SA facial characteristics which would allow the development of a South African RFTP. The Korean studies on respirator fit correspond well with our findings where they identified that the Korean worker population differed significantly from the American population used to design respirators. Based on this the Koreans developed their own test panels and began the design of masks specifically for Korean workers [6,14].

## Conclusions

The large percentage of failed fit tests indicates that reliance on medium respirators for all workers is likely to be a major problem in South Africa.

Larger studies are needed on respirator fit and facial dimensions in the South African workforce to ascertain if the findings of this study are applicable to the general working population.

Ideally respiratory protective equipment should not be use as the primary control to protect workers against airborne hazards. From this limited study it was shown that a fit test programme is essential before issuing respiratory protective equipment and more than one type and size respirator should be included in any programme as clearly "one size does not fit all".

## List of Abbreviations

PPE: Personal protective equipment; OSHA: Occupational Safety and Health Administration; USA: Unite States of America; RFTPs: Respirator Fit Test Panels; NIOSH: National Institute for Occupational Safety and Health.

## Competing interests

The authors declare that they have no competing interests.

## Authors' contributions

AS conceived of the study, carried out the respirator fit testing, participated in the design of the study and drafted the manuscript. KW conceived of the study, and participated in its design, performed the statistical analysis and help drafting the manuscript. RF participated in its design and helped to draft the manuscript. All authors read and approved the final manuscript.
